# Lunate-capitate arthrodesis for scaphoid nonunion: a comparative study

**DOI:** 10.1186/s12891-024-07755-w

**Published:** 2024-08-20

**Authors:** Amr Elshahhat, Yaser Abed, Khaled Nour

**Affiliations:** Orthopedic Surgery Department, Mansoura University, Algomhoria Street, Mansoura, 33516 Dakahlia Egypt

**Keywords:** Scaphoid nonunion, Lunate-capitate fusion, Four-corner fusion, Wrist arthritis

## Abstract

**Background:**

Scaphoid nonunion advanced collapse (SNAC) injuries are frequently associated with irreversible degenerative wrist arthritic changes that necessitate surgical intervention. Midcarpal fusion remains the mainstay of the management of SNAC II and III injuries. A successful four-corner fusion (4CF) relies on a stable lunate-capitate fusion (LCF). There have been reports of management relying solely on LCF. The outcomes of LC- and 4 C-fusions in SNAC injuries were not widely documented. The objective of this research is to provide valuable insights into the effectiveness of both fusion procedures in the management of SNAC II and III wrist injuries, with a focus on reporting associated complications, functional and radiological outcomes.

**Patients and methods:**

This retrospective study encompassed 65 patients diagnosed with SNAC II and III wrist injuries who underwent limited wrist fusion procedures between 2015 and 2024, with a minimum of 2 years of postoperative follow-up. Exclusion criteria encompassed patients with carpal instability, prior wrist surgical interventions, and scapholunate advanced collapse. Following the fusion procedure performed, patients were stratified into two groups: the LCF group consisting of 31 patients, and the 4CF group comprising 34 patients. Preoperative and intraoperative data were retrieved from the patient’s medical records. At their final follow-up appointments, patients underwent comprehensive radiographic and clinical evaluations. Clinical outcomes including hand grip strength, range of motion, the Disabilities of the Arm, Shoulder, and Hand Score, and the Mayo Modified Wrist Score, were compared between groups. Any associated complications were reported.

**Results:**

The average healing time was 74.7 ± 15.6 and 72.2 ± 13.2 days for the LCF and 4CF groups, respectively. At the final visit, all patients showed functional improvement relative to their preoperative status, with comparable wrist range of motions observed in both groups. The functional wrist scores were slightly better in the LCF patients (*P* > 0.05). The average grip strength was significantly greater in the LCF group (*P* = 0.04), with mean strength values of 86.8% and 82.1% of the contralateral side, for the LCF and 4CF groups, respectively.

**Conclusion:**

The LCF is not less efficient than the 4CF in the treatment of SNAC II and III wrist injuries. Through a less time-consuming procedure, LCF can efficiently provide comparable results to 4CF.

**Level of evidence:**

level IV evidence.

## Background

Scaphoid nonunion advanced collapse (SNAC)- injuries are usually associated with variable degree of wrist degenerative wrist osteoarthritis that necessitates surgery [1]. This subsequent wrist osteoarthritis demands partial or total scaphoidectomy combined with midcarpal joint stabilization. Two surgical options have been put forth: central column stabilization through lunate-capitate fusion (LCF) in conjunction with triquetrum preservation or excision, or fusing the lunate, triquetrum, capitate, and hamate bones (four-corner fusion) [2].

Four-corner fusion (4CF) had been favored for treating SNAC injuries due to its increased surface area of the bony union and lower nonunion rate. Still, current studies have demonstrated that LCF provides good functional outcomes, especially in light of recent surgical implant developments [3]. The literature reports limited data comparing LCF and 4CF in SNAC wrist injuries. In addition to reporting related complications, this study sought to compile both strategies for managing SNAC II and III conditions regarding functional and radiological outcomes.

## Patients and methods

**Fig. 1:**
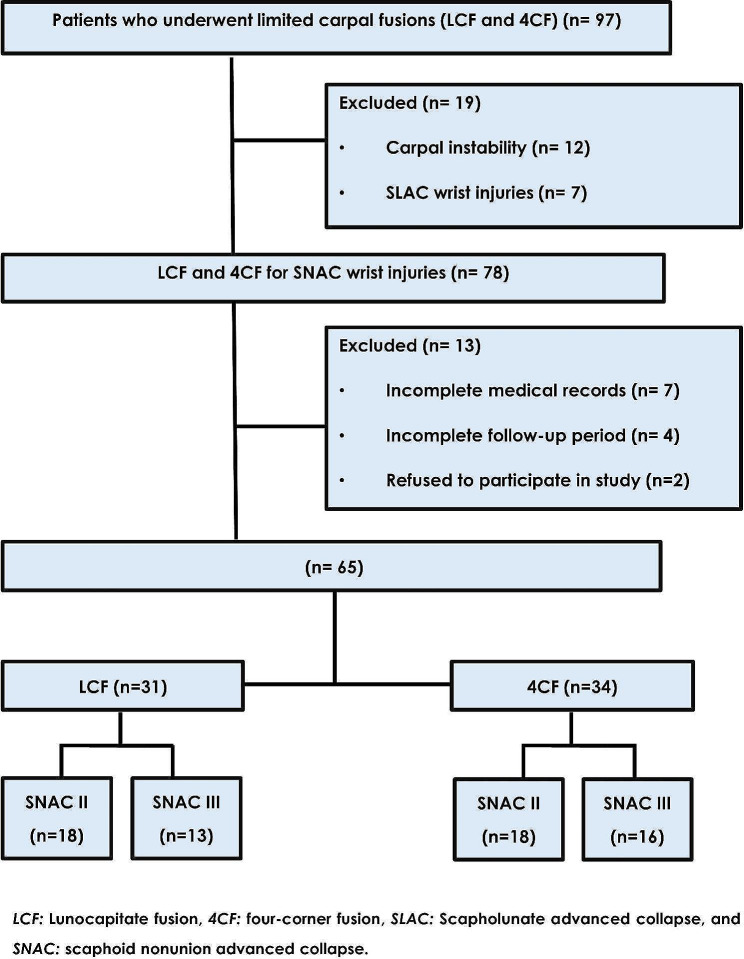
The flowchart of the patient enrollment process

This study was approved by the ethics committee of our institution following the Declaration of Helsinki’s principles. The medical records of every patient who had LCF or 4CF were retrieved between 2015 and 2024. Patients who underwent either surgery for SNAC II or III wrist injuries with at least a two-year follow-up were included in this study. Patients with previous wrist surgeries and those who underwent either surgery for scapholunate advanced collapse (SLAC) or carpal instability were excluded. All study’s participants were informed about other surgical options, such as proximal row carpectomy (PRC). They received adequate information regarding the potential advantages and disadvantages of PRC or limited fusion procedures regarding postoperative range of motion (ROM), hand grip strength, and possible complications. It is worth mentioning that wrist arthroplasty is not a viable option at our institution. Written consent was obtained from patients included in this study. Figure 1 shows the flowchart for the patient enrollment procedure.

SNAC II and III wrist injuries were identified in 65 patients. Patients who had LCF included 27 men (87%) and 4 women (13%). In twenty-one patients (67.7%), the affected wrist was the right one. The mean age of the patients was 31.5 ± 5 years (range: 23–40). 20 patients (64.5%) had their dominant hand affected. There were 22 manual laborers (71%), 5 students (16%), and 4 housewives (13%), who represented the patients in this group. The second group of patients underwent 4CF; they included 32 males (94.1%) and 2 females (5.9%). The right wrist was injured in 23 patients (67.6%). The mean age of the patients was 35.6 ± 9 years (range: 20–58). Most of these patients (82.3%) were manual laborers. Twenty-five patients (73.5%) sustained an injury to their dominant hand. Table 1 presents patients’ demographics.

**Table 1:** Patients’ demographics in LCF- and 4CF-groups

Demographics	LCF-group (*n* = 31)	4CF-group (*n* = 34)	*P*-value
Age	31.5 ± 5.3 years	35.6 ± 9.5 years	0.19
Gender	Males (n = 27; 87%) Females (n = 4; 12.9%)	Males (n = 32; 94.1%) Females (n = 2; 5.8%)	
Side	RT (n = 21; 67.7%) LT (n = 10; 32.2%)	RT (n = 23; 67.6%) LT (n = 11; 32.4%)	
Dominance	Dominant (n = 20; 64.5%) Non-dominant (n = 11; 35.4%)	Dominant (n = 25; 73.5%) Non-dominant (n = 9; 26.4%)	
Occupation	ML (n = 22), ST (n = 5), HW (n = 4)	ML (n = 28), HW (n = 2), ST (n = 3), SP (n = 1)	
SNAC Stage	Stage II (n = 18; 58%) Stage III (n = 13; 41.9%)	Stage II (n = 18; 52.9%) Stage III (n = 16; 47%)	
Scaphoid nonunion duration	4.9 ± 1.9 years	6.8 ± 2.9 years	0.0096*
Operative time	80.4 ± 6.2 min	116.4 ± 10.7 min	0.0001*
Follow-up period	38.4 ± 6.7 months	46 ± 26.2 months	0.59

(*) indicates statistically significant. (SD; standard deviation, n; number, RT; right, LT; left, ML; manual laborer, ST; student, HW; housewife, SP; sport, and SNAC; scaphoid nonunion advanced collapse)

The wrist active ROMs, Disabilities of the Arm, Shoulder & Hand (DASH) score [4], the Mayo Modified Wrist (MMW) score [5], the visual analog scale (VAS) pain grade [6], and the hand grip strength in kg using the Baseline^®^ hydraulic hand dynamometer (Fabrication Enterprises, Elmsford, NY, U.S.A.) demonstrated the preoperative status for the patients. Detailed functional status of LCF- and 4CF-patients are shown in Tables 2 and 3.

**Table 2:** Pre- and postoperative wrist active ROMs in LCF- and 4CF-patients

Wrist ROMs	Preoperative			Final follow-up		
	LCF-group (*n* = 31)	4CF-group (*n* = 34)	*P* value	LCF-group (*n* = 31)	4CF-group (*n* = 34)	*P* value
	(Mean ± SD)			(Mean ± SD)		
Flexion ROM	45.3 ± 13.3º	46.4 ± 12.5º	0.8	52.9 ± 9.9º	52.9 ± 9.8º	0.49
Extension ROM	41.1 ± 9.7º	41.2 ± 9.4º	0.92	55.3 ± 11.5º	54.1 ± 9.3º	0.32
Flexion-extension arc	86.9 ± 22.1º	87.8 ± 20.1º	0.43	108 ± 19.7º	107 ± 17.4º	0.4
Flexion-extension arc percent	66.6 ± 12.6%	67.4 ± 12%	0.99	88.5 ± 7%	86.2 ± 8%	0.28
Ulnar deviation ROM	25 ± 6.15º	24.4 ± 5.18º	0.33	27.6 ± 4.2º	28.1 ± 3.7º	0.87
Radial deviation ROM	11.1 ± 2.4º	11.3 ± 2.5º	0.37	12.3 ± 1.9º	12.1 ± 2.3º	0.77
Radio-ulnar arc	37.7 ± 4.5º	36 ± 5.1º	0.08	40.1 ± 4.6º	40.4 ± 4.4º	0.37
Pronation ROM	75 ± 5º	74 ± 4.47º	0.18	79.9 ± 4.4º	79.2 ± 3.8º	0.39
Supination ROM	72 ± 5.2º	72.2 ± 4.1º	0.68	74.8 ± 4.6º	76.1 ± 4.4º	0.28
Pronation-supination arc	146.5 ± 7.2º	145 ± 7.2º	0.26	154.7 ± 7.9º	155.3 ± 7º	0.38

(SD; standard deviation)

**Table 3:** Pre- and postoperative functional status in LCF- and 4CF-patients

Functional status	Preoperative			Final follow-up		
	LCF-group (*n* = 31)	4CF-group (*n* = 34)	*P* value	LCF-group (*n* = 31)	4CF-group (*n* = 34)	*P* value
	(Mean ± SD)			(Mean ± SD)		
MMWS	44.1 ± 20.7	41.5 ± 20	0.29	79.8 ± 10.3	76.6 ± 9.9	0.23
Grip strength	15.4 ± 6 kg	14.1 ± 5.6 kg	0.18	25.7 ± 3.6 kg	24.3 ± 3.2 kg	0.18
Grip strength ratio (percent)	53.4 ± 21.1%	49.1 ± 19.9%	0.202	86.8 ± 11%	82.1 ± 11%	0.045*
VAS score	5.6 ± 1.8	6 ± 2	0.19	0.58 ± 0.7	0.67 ± 0.8	0.72
DASH score	57.1 ± 14	54.7 ± 15.1	0.25	13.7 ± 4.3	14.7 ± 4.6	0.18

(*) indicates statistically significant. (SD; standard deviation, MMWS; Mayo Modified Wrist Score, VAS; visual analog scale, DASH; Disabilities of the Arm, Shoulder, and Hand Score)

### Surgical procedures

Following general or supraclavicular regional anesthesia, patients were placed in the supine posture. Routinely, antibiotic prophylaxis was administered ten minutes before exsanguination of the limb and inflation of a tourniquet. The dorsal wrist approach [7] was utilized in all instances, with eight to ten-centimeters-skin incision made in line with the third metacarpal axis and centered on the wrist joint. The overlying retinaculum of the third compartment enclosing the extensor pollicis longus (EPL) tendon was incised, and the tendon was retracted radially. The tendons within the fourth compartment were retracted to the ulnar side following the incising of the overlying fascia. A 2 cm portion of the posterior interosseous nerve’s terminal branch was taken down, with the proximal end cauterized. As a result, the wrist capsule was exposed and incised with a radial-based flap that could be readily reflected and lifted away from the triquetrum.

**Fig. 2:**
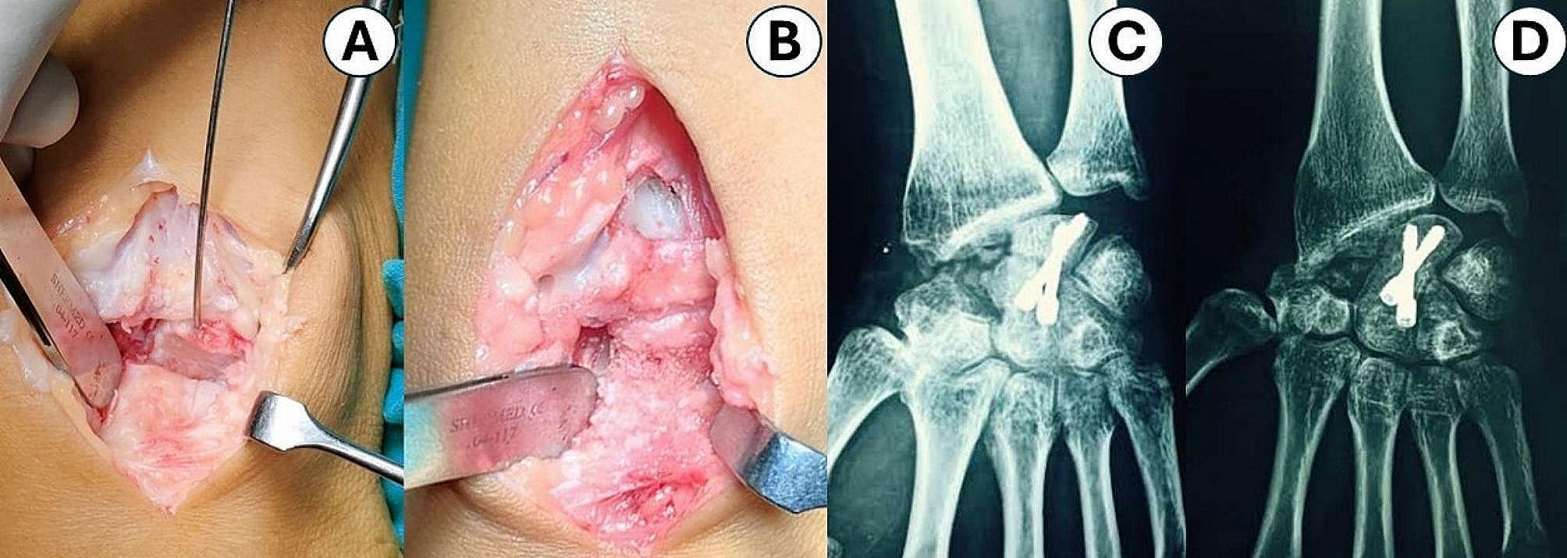
**A**: Intraoperative clinical photo demonstrating preparation of the LC articulation site, **B**: The LC site following fixation and bone grafting, **C**: Early postoperative wrist radiograph showing complete scaphoidectomy and LCF using two Herbert screws inserted in a retrograde manner; **D**: Wrist radiograph showing healing of the LCF site

In the LCF-group (*n* = 31); total scaphoidectomy was carried out with extreme caution to preserve the volar radiocarpal ligaments. Radial styloidectomy was performed in 9 patients (29%) to prevent wrist impingement on radial deviation. Then the next step was to prepare the fusion site (Fig. 2) by denuding the articulation of the lunocapitate (LC) joint using an 11 mm-scalpel knife, and a small high-speed burr. Afterward, as a joystick, a 1.5 mm-Kirshner wire (K-wire) was advanced into the lunate to correct its extension. So that the lunate overhung and coaptated the capitate. In a retrograde fashion, a 1.5 mm-K-wire was advanced from capitate to lunate as a temporary LC stabilization. The position of the LC K-wire was checked by fluoroscopy. Accordingly, the joystick lunate K-wire could be removed since it was no longer necessary. Currently, two serrated guide wires were advanced in an antegrade manner (lunate to capitate path) in 14 out of 31 patients, and in a retrograde manner (capitate to lunate path) in 17 out of 31 patients, parallel to the LC longitudinal axis. Afterward, 3 mm Herbert headless compression screws (HCS) (Synthes^®^) of the desired size were inserted compressing the LC fusion site (Fig. 2) In 27 cases, two screws were used to compress the LC articulation, and in 4 cases, three screws. Making sure that, depending on where the screws access the bone, the heads of the inserted screws become subchondral beneath the articular surface of the lunate or the capitate. Maximal wrist flexion encouraged the screws to advance while using lunate-capitate directed screws. A cancellous bone was prepared for grafting in six patients. Among them; in four instances, from the removed scaphoid, and in two cases, from the removed scaphoid and distal radius.

In the 4CF-group (*n* = 34); Patients underwent the surgical procedure previously mentioned, which entailed scaphoidectomy (*n* = 13) and preparation of the LC fusion site using the same mentioned technique. In addition, capitohamate, triquetrohamate, and lunotriquetral joint fusion sites were prepared in the same manner. Afterward, one 3 mm-headless compression screw was advanced in an antegrade manner to achieve LCF. Another appropriately sized screw was inserted from the triquetrum through the hamate to capitate compressing the three bones together. Cancellous grafts from the distal radius and excised scaphoid were harvested at fusion sites in 25 patients (73.5%). The capsulotomy was repaired, extensor tendons were relocated, and the extensor retinaculum was approximated. The skin incision was closed. The operative time was reported from the skin incision to wound closure. In ten-degree extension, the wrist was immobilized in a below-elbow splint for 8 weeks. ROM exercises started afterward to regain ROM and hand grip strength.

The union at the fusion site was evaluated on radiographs (three views) without requiring CT scans. Wrist radiographs were taken at three and six weeks, and then every three weeks, until fusion is achieved. The radiographic healing time was reported in weeks. Bridging trabeculae at the fusion sites without any direct tenderness over the fusion site was considered healing. The healing was initially observed by the operating surgeon. Subsequently, this healing was corroborated by two hand surgeons who were not involved in the research study. The fusion was deemed to be successfully healed only upon confirmation from both independent surgeons. At the last visit, the clinical status was evaluated as per hand grip strength and the DASH, MMW, and VAS scores. Using an orthopedic goniometer, the ulnar-radial, flexion-extension, and pronation-supination arcs were demonstrated in degrees. Any LCF- or 4CF-related complications were documented.

Data was fed into a computer and analyzed using IBM SPSS Corp. IBM SPSS Statistics 22.0 for Windows. NY / Armonk: IBM Corp. The qualitative data were described in terms of numbers and percentages. The median (minimum and maximum) was used to characterize non-parametric quantitative data, whereas the mean and standard deviation were used to characterize parametric data. The normality of the data was assessed using the Kolmogorov-Smirnov Test. The t-test was applied to compare two independent groups with normally distributed data. Given two independent groups with abnormally distributed data, the Man-Whitney U test was employed to compare the groups. The results were assessed for significance at the 0.05 level.

## Results

Patients completed an average follow-up period of 42.3 ± 19.7 months; among whom, 31 had LCF and 34 had 4CF. The length of scaphoid nonunion was significantly longer in the 4CF group (*P* = 0.0096). In each group, eighteen patients suffered from SNAC II injuries. whereas SNAC III was present in 47% and 41.9% of the 4CF and LCF groups, respectively. With an average difference of 36 ± 4 min, the mean operative time was shorter in the LCF-group (P˂0.05). Complete radiographic union was observed in 96.7% (30/31) of LCF patients and 94.1% (32/34) of 4CF patients, with average healing times of 74.7 ± 15.6 and 72.2 ± 13.2 days for the respective LCF and 4CF groups (*P* = 0.23). Although non-grafting groups with either 4CF or LCF had a longer mean healing time, there was no significant difference (Table 4)

**Table 4:** Radiographic healing time in LCF- and 4CF-groups

Fusion group	Healing time (Mean ± SD)		*P* value
**LCF-group** (*n* = 31)	72.2 ± 13.2 days		
	Grafting group (*n* = 6)	68.3 ± 12.9 days	*P* = 0.218
	No grafting (*n* = 25)	73.1 ± 13.4 days	
**4CF-group** (*n* = 34)	74.7 ± 15.6 days		
	Grafting group (*n* = 25)	74.7 ± 15.5 days	*P* = 0.48
	No grafting (*n* = 9)	75 ± 17.1 days	

All patients showed functional improvement at the final visit compared to their preoperative status. The LCF group’s patients had slightly higher average flexion-extension ROM, but this difference was not statistically significant. They also showed comparable average radio-ulnar and pronation-supination ROMs to 4CF-group. The MMW, DASH, and VAS scores of patients in either group were comparable. The mean postoperative grip strength ratio revealed notable difference between fusion techniques (*P* = 0.04). For the LCF- and 4CF-groups, the corresponding mean strength was 86.8% and 82.1% of the contralateral normal side. An average of 1.4 kg of strength was reflected by this 4.7% difference. Tables 3 and 4 demonstrate structured preoperative and postoperative clinical data for patients in both groups.

**Fig. 3:**
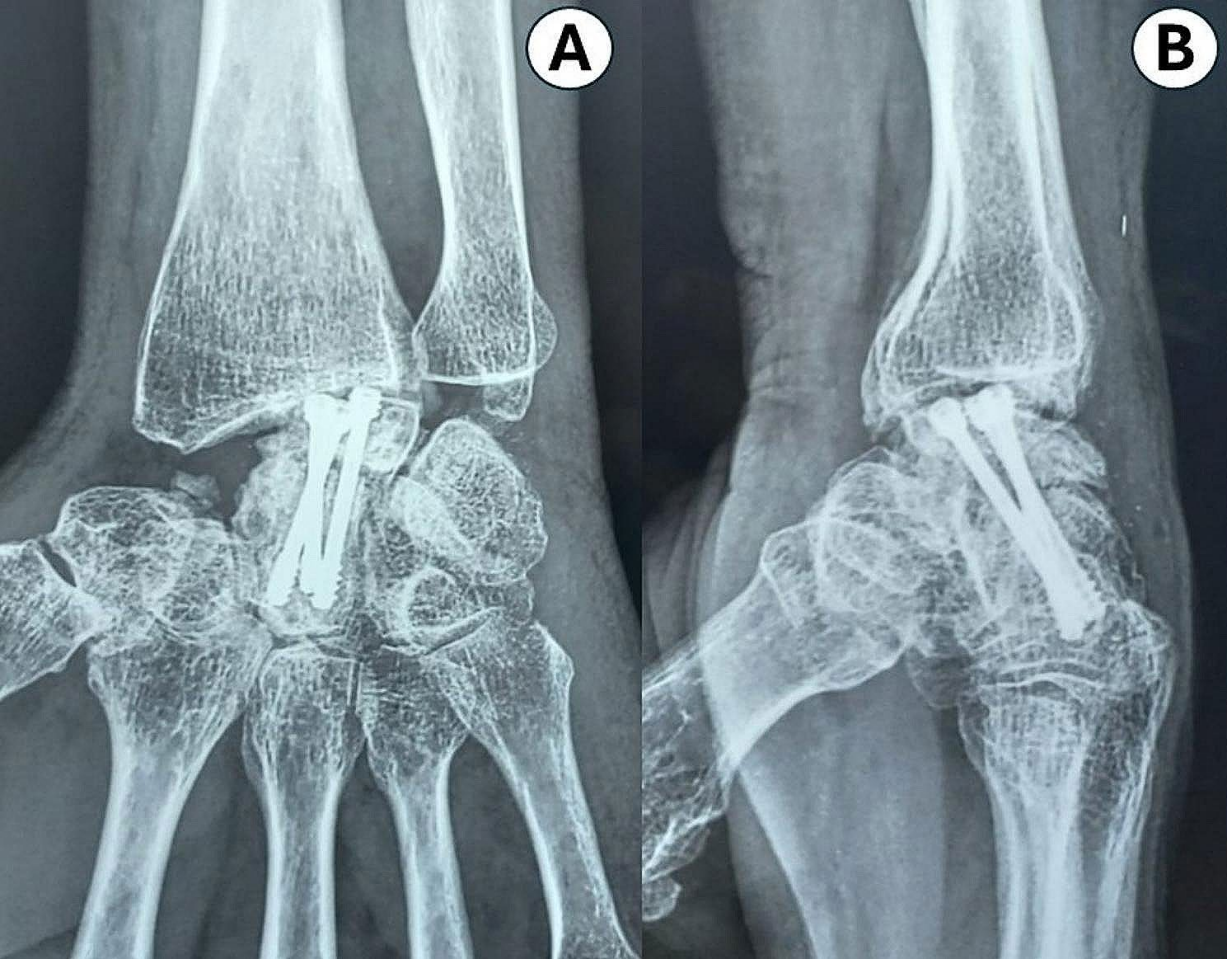
**A**: Anteroposterior, and **B**: Lateral wrist radiographs demonstrating RL osteoarthritis with backed out screws of the lunate following LCF with antegrade inserted Herbert screws

Four patients from the 4CF-group and another from the LCF-group developed complex regional pain syndrome, all received conservative treatment and improved clinically. Two patients—one from each fusion group—experienced ulnar-side wrist pain, and both declined further care. Nonunion of fusion site was noted in three cases (one after LCF, and the other two following 4CF); in all three, wrist fusion had been performed. LCF nonunion associated backing out of screws with subsequent radio-lunate (RL) wrist arthritis (Fig. 3); this patient complained of radial side wrist pain that worsens with ulnar deviation and wrist flexion. Additionally, five patients with 4CF and six patients with LCF had RL osteoarthritis. All of them—aside from the previously mentioned patients—were asymptomatic and showed complete healing of fusion site and proper positioned screws. They never experienced wrist pain; however, three of them demonstrated ulnar translocation of carpus on final radiographs. There was no infection in either fusion group, even though there was a noticeable difference in the mean operative time between them.

## Discussion

Without treatment, SNAC wrist injuries are often associated with degenerative wrist arthritic changes that are inevitable and produce chronic pain, necessitating surgery [8]. Vender et al. demonstrated the three consecutive stages of arthritis: stage I involved arthritis between the injured scaphoid’s distal pole and the radial scaphoid fossa, stage II had arthritis between the injured scaphoid’s proximal fragment and the capitate, and stage III involved arthritis of the scaphocapitate, LC, and radio-scaphoid interfaces [9]. Even in more advanced instances, RL articulation is typically preserved in SNAC [10]. Hence, limited wrist fusions are now a commonly utilized surgical technique for treating patients with localized wrist arthritis. Limited carpal fusion might be accomplished with either LCF or 4CF [11–14].

The 4CF attempts to restore the carpus alignment with a painless mobile joint in the treatment of SNAC injuries [15–20]. The reported revision procedures and nonunion rate account for 14.3% of 4CF [21]. The cornerstone of a good 4CF is represented in a steady LCF. The idea of treating such injuries with isolated LCF rather than 4CF was raised by this biomechanical proposition [22].

There isn’t much data in the literature comparing the outcome of both fusion strategies. According to Schriever et al. [23], there were no significant differences in grip strength ratio between the two methods (69% and 79% of the contralateral side for LCF and 4CF, respectively). Ferreres et al. and Andronic et al. reported a mean grip strength ratio of 68% after 4CF and 66–71% after LCF, in their retrospective case studies [24, 25]. Similarly, non-significant variations in grip strength were also noted by Duraku et al. [26] and Gaston et al. [27], with higher values following 4CF. In this investigation, however, it was found that the grip strength ratio increased with LCF compared to 4CF, with mean strengths of 82.1 ± 11% and 86.8 ± 11%, respectively (*P* = 0.045). Besides, the hand grip strength (N) did not reveal any significant difference between either fusion procedure (*P* = 0.18). The grip strength ratio calculation method (the strength of the operated hand divided by the strength of the contralateral sound hand) might be the cause of this discrepancy. Given that the operated hand was predominantly the dominant hand for most patients in both procedures (64.5% of LCF patients and 73.5% of 4CF patients), this dominance could have played a role in both the observed significance of the grip strength ratio (%) and the lack of significance of the grip strength (N). Another critical consideration is the necessity of adjusting measurements for right-handed patients, as the right upper limb frequently exhibits a strength advantage of approximately 10% [28]. It is noteworthy that in the context of this study, no such correction was applied for the grip strength assessments of right-handed patients. This oversight may have implications for the interpretability of the results, as it might fail to account for inherent asymmetries in muscle strength that are prevalent among right-handed individuals.

This retrospective study found no relevant differences in ROM between 4 F and LCF, as reported by antecedent studies [23, 26, 27]. Our study did not find any significant differences in functional status, as illustrated by previous studies [23, 26, 27]. Nevertheless, the functional outcomes for SLAC injuries were included in the aforementioned studies [23, 26, 27]. Reports from previous studies, including case series and comparative studies, on the management of SNAC wrist injuries using the LCF procedure are presented in Table 5. Compared to 4CF, the LCF is thought of as a more limited surgical procedure. This study showed that the mean operative time for LCF was 36 ± 4 min less than that of 4CF (*P* < 0.05), as reported by previous studies [26, 27, 29]. Reduced soft tissue dissection and, consequently, minimal tissue scarring can be achieved by limiting the number of fused intercarpal joints [29, 30].

**Table 5:** LCF in SNAC wrist injuries: demographics and results from previous research (case series and comparative studies)

Study/ Design / Indications		(*N*) (M-F)	Age (years)	Follow-up (months)	Dominant hand	Concomitant procedure	Fixation device	Bone Graft	Radiographic healing time (days)	Fusion rate	PROs	Post-operative protocol	Complications
Goubier [14], 2007 Case series/ SNAC		13 (M-F: 12 − 1)	48 (36–61)	29 (27–36)	NM	Scaphoidectomy ± Triquetrum excision	2 HCS	None	63 (42–91)	12/13 (92%)	Mayo score NM Flex-Ext arc 64° VAS score 1.25 (1–5) Grip strength 22 kg (15–35 kg)	Immobilization in a splint for 1 week, ROM exercises started afterward	RSD (1 case) Nonunion (1 case)
Giannikas [13], 2010 Case series/ SNAC		8 (M-F: 8 − 0)	39 (29–52)	52 (24–83)	6 (75%)	Proximal pole scaphoid excision ± Distal pole SC fusion ± Radial styloidectomy	2 HCS	Iliac graft (8 cases)	NM	8/8 (100%)	Mayo score 69.7 (58.7–75) Flex-Ext arc 83.5° (63–90°) VAS score 1.25 Grip strength 43 kg (34–54 kg)	Immobilization in short arm cast for 8 weeks, ROM exercises started afterward	NO
Hegazy [37], 2015 Case series/ SNAC		12 (M-F: 9 − 3)	44 (28–66)	37.4 (12–47)	7 (58.3%)	Scaphoidectomy	2 HCS	Local graft from carpus (4 cases)	70 (60–90)	12/12 (100%)	Mayo score 81.7 (75–90) Flex-Ext arc 89.3° (78–99°) VAS score 1 (0-0.05) Grip strength 40.3 kg (31–46 kg)	Immobilization in a short arm splint for an average of 48 days.	NO
**Comparative studies (LCF versus 4CF)**													
Duraku [26], 2022 Prospective Cohort/ / SNAC, SLAC	LCF	34 (M-F: 26 − 8)	60 ± 11	Clinical (12) Complications (42-63.6)	26 (77%)	Scaphoidectomy ± Triquetrum excision (LCF) ± Radial styloidectomy	HCS or K wires (23.5%)	Autograft from scaphoid	NM	28/32 (87.5%)	PRWHE score 48 points Grip strength 21 (19–25) Flexion 35°	Removable short cast after 5 days, ROM exercises between 2 and 6 weeks, Physiotherapy after 3 months (radiographic confirmation of fusion).	Nonunion: LCF, (6) 4CF (3) Impingement: LCF, (3) 4CF (4) Material failure: LCF, (1) 4CF (1) Re-operation: LCF, (10) 4CF (7)
	4CF	29 (M-F: 28 − 10)	58 ± 10		15 (52%)		HCS (31%), dorsal plate (62%), K wires (7%)			26/29 (89.6%)	PRWHE score 46 points Grip strength 18 (14–31) Flexion 24°		
Schriever [23], 2023 RCT/ SNAC, SLAC	LCF (n = 32)	64 (M-F: 54 − 10)	60 (39–79)	12	Dominant side was operated to same extent in both groups	Scaphoidectomy	K wires	LCF: Autograft from scaphoid 4CF: Autograft iliac crest or radius	NM	30/32 (93.7%)	Flexion 20 ± 14º Extension 34 ± 13º Key pinch 69 ± 22º Grip strength ratio 69 ± 22%	wrist immobilization in a short arm plaster cast until radiological evidence of bone fusion, Active wrist mobilization afterward, Physiotherapy after K-wires removal	Rupture of extensor tendons LCF, (2) 4CF (1) Superficial wound Infection: LCF, (1) 4CF (1) Deep infection: 4CF (1) Nonunion: LCF, (2) 4CF (4) Dorsal tilt of lunate: LCF, (7) 4CF (7)
	4CF (n = 32)									28/32 (87.5%)	Flexion 21 ± 14º Extension 38 ± 14º Key pinch 87 ± 18º Grip strength ratio 79 ± 20%		
Current study Retrospective/ SNAC	LCF	31 (M-F: 27 − 4)	31.5 ± 5.3	38.4 ± 6.7	20 (64.5%)	Scaphoidectomy ± Radial styloidectomy	2 HCS	Local graft from excised scaphoid ± distal radius LCF (6) 4CF (25)	72.2 ± 13.2	30/31 (96.7%)	Mayo score 79.8 ± 10.3 Flex-Ext arc 108 ± 19.7º VAS score 0.58 ± 0.7 Grip strength ratio 86.8 ± 11%	Immobilization in a below-elbow splint for 8 weeks, ROM exercises started afterward	RSD: LCF, (1) 4CF (4) Ulnar-side wrist pain: LCF, (1) 4CF (1) Nonunion: LCF, (1) 4CF (2) RL osteoarthritis: LCF, (6) 4CF (5)
	4CF	34 (M-F: 32 − 2)	35.6 ± 9.5	46 ± 26.2	25 (73.5%)				74.7 ± 15.6	32/34 (94.1%)	Mayo score 76.6 ± 9.9 Flex-Ext arc 107 ± 17.4º VAS score 0.67 ± 0.8 Grip strength ratio 82.1 ± 11%		

(LCF: Lunate-capitate fusion, 4CF: Four corner fusion, N: number; M-F: Male-Female, NM: Not mentioned, SC: scaphocapitate, HCS: Headless compression screw, PROs: Patient-reported outcomes, VAS: Visual analog score, PRWHE score: Patient-Rated Wrist Hand Evaluation score, RSD: reflex sympathetic dystrophy, RL: radio-lunate)

The literature detailed different fusion devices, including K wires [18]; staples [31]; spider dorsal plates [15]; bioabsorbable plates [32]; screws [17]; or cerclage [33]. For LCF and 4CF, the two previous comparison studies used various fixation devices. Gaston et al. employed two antegrade headless compression screws (HCS) in the LCF, while K-wires, staples, or screws were utilized for the 4-CF, [27]. In their investigation, González Porto et al. used one antegrade HCS for LCF, and screws, staples, or spider plates for 4CF [2]. In this study, both fusion techniques utilized HCSs. The employed implant may have a detrimental impact on the fusion site’s healing. Reportedly, for 4CF, the nonunion rate was 16–62% for dorsal plates and 3–18% for K wires or staples [34, 35]. Ronchetti et al. reported a nonunion rate of 33–50% in LCF using K wires and staples [36]. The use of HCSs considerably reduced the nonunion rate. Antecedent investigations reported a zero nonunion rate after LCF with HCSs [27, 37]. As noted in this study, Healing of LC articulation was not impacted by the number of utilized screws (≥ 2 screws in LCF, single screw for 4CF). Similarly, González Porto et al.. reported a 0% nonunion rate with a single screw [2]. Our results revealed that the screw’s trajectory did not affect the fusion site’s potential to heal. This is consistent with what Yao et al. [38] previously reported. Contrasting the healing outcomes of harvesting cancellous bone grafts at the fusion site from the excised scaphoid alone or both the excised scaphoid and distal radius, there was no obvious difference in this study. The fusion site’s capacity to heal in the LCF and 4CF was not influenced by either the application or avoidance of grating. In a similar vein, previous studies correlated nonunion rates to the method of fixation used rather than augmentation bone grafting [2, 39, 40].

All patients in this study underwent total scaphoidectomy with triquetrum preservation. It is still debatable whether the triquetrum bone should be sacrificed during LCF. According to several studies, the triquetrum sacrifice during LCF was correlated to a lower risk of RL contact pressure and ulnar-side wrist pain [24, 27, 29, 30], it might also facilitate intraoperative lunate repositioning [27]. Advocates of triquetrum preservation also declined its association with postoperative ulnar-sided wrist discomfort employing limited soft tissue dissection and intact proprioception of the radio-triquetral ligaments [24, 37, 41]. Two individuals in our study (one in each group) had ulnar-side wrist pain. Even though both patients’ fusion sites were fully consolidated, the definitive explanation for their wrist pain could not be precisely determined. We suggested that during total scaphoidectomy, a potential intraoperative injury to the volar radio-carpal ligaments, including the long RL ligament and radio-scapho-capitate ligament, might be affronted of future wrist pain through translocation of the remaining carpus. Accordingly, as the antecedent report stated [42], it is not always required to remove the scaphoid’s distal pole. This may limit the injury to the volar ligaments that are still attached to the remaining scaphoid.

Ferreres et al. found a 25% incidence of postoperative RL osteoarthritis following LCF; nevertheless, all patients exhibited no clinical symptoms [24]. In this study, five patients following 4CF and six following LCF had RL osteoarthritis. All of them, apart from the patient who had LCF nonunion with backed out screw, had their triquetrums preserved and were clinically free. We postulate that the risk of intraoperative articular manipulation and damage during the advancement of the LC screw(s) may be related to the incidence of postoperative RL osteoarthritis. Given that two or three screws are advanced during LCF rather than just one during 4CF, it may be clear why the percentage of osteoarthritis with LCF (22.5%) is higher than that of cases with 4CF (14.7%).

A variety of studies [30, 37, 41, 43, 44] utilized the LCF and demonstrated a considerable union rate of 93.5% [24, 27, 45]. Nevertheless, the necessity of revision procedures remains unclear. There is still uncertainty regarding the long-term viability of 4CF and LCF surgeries [25]. It remains difficult to arrive at assumptions from prior findings alone because of the broad variety of outcome metrics and surgical procedures reported in the literature. The retrospective nature of this study remains the main point of weakness. Besides, it is critical to recognize two important confounding variables in this study: the length of the scaphoid nonunion and the number of screws used to fuse the LC articulation. Using multiple screws could accelerate the healing of the LC fusion in the LCF group, which could bias the results in favor of this surgical strategy. On the other hand, the 4CF group’s results might be negatively influenced by the noticeably longer nonunion duration. The results of this study should therefore be interpreted cautiously. Future research should focus on elucidating these confounding variables to provide a more comprehensive understanding of their clinical and radiological impact across different surgical techniques. However, the study strengths are represented in the large number of included patients, with a considerable follow up period, and utilizing validated scoring systems. Future randomized controlled trials may yield far more substantial results and guide for more solid recommendations.

## Conclusion

In the treatment of SNAC II and III wrist injuries, the LCF is not less efficient than the 4CF. Through a less time-consuming procedure, LCF can efficiently provide comparable results to 4CF.

## Data Availability

No datasets were generated or analysed during the current study.
